# Male Depression – Are gender-related personality traits associated with the severity of depressive symptoms?

**DOI:** 10.1192/j.eurpsy.2025.858

**Published:** 2025-08-26

**Authors:** V. Rößner-Ruff, J. Krieger, K. Friedrich, C. A. Penkov, M. Ziegenbein, F. Führmann, I. T. Graef-Calliess

**Affiliations:** 1 Research and Development, Wahrendorff Clinic, Sehnde; 2 Research and Development, KRH Psychiatry Wunstorf, Wunstorf; 3 Clinic for Psychiatry and Psychotherapy, ZfP Südwürttemberg, Ravensburg, Germany

## Abstract

**Introduction:**

Higher prevalence & incidence rates of depressive disorder in women compared with men are among the recurring findings from epidemiologic & clinical studies. The literature suggests that this is not due to a lower need for treatment of depression in men. In the context of the concept ‘male depression’ (MD), it is often argued that men tend to have so called ‘non-typical’ depressive symptoms. These symptoms (such as aggressiveness, irritability, alcohol use, risk-taking & antisocial behavior) are rarely considered in the diagnosis of depressive disorders, which may lead to underdiagnosis. With regard to the occurrence of ‘non-typical’ depressive symptoms and the concept MD, the importance of masculine personality traits is often discussed. The topic of the session is the extent to which gender-related personality traits such as masculine, feminine, androgynous & undifferentiated are associated with the occurrence and severity of ‘non-typical’ and ‘typical’ depressive symptoms in women and men with a depressive disorder.

**Objectives:**

The results of a study in a clinical setting will be presented. The aim of the study was to investigate whether above mentioned gender-related personality traits are associated with increased severity of depressive symptoms and whether there are differences between women & men.

**Methods:**

Depressive symptoms (GMDS & BDI II) and gender-related personality traits (GEPAQ) were assessed in female & male patients (≥ 18 years) with an unipolar depressive disorder (ICD-10). Participants were recruited from inpatient settings & day clinics of specialized psychiatric-psychotherapeutic hospitals in Germany. The multicenter study with a cross-sectional design has been completed. Data from the clinical sample were analyzed using multiple linear regression analysis.

**Results:**

Multiple linear regression analysis: criteria variable **GMDS** & predicting variable **GEPAQ** (**masculine** pt) b-coefficient women = -1.36 (n.s.) & b-coefficient **men** = **-1.48** (p ≤ .01); criteria variable GMDS & predicting variable GEPAQ (feminin pt) b-coefficient women = .12 (n.s.) & b-coefficient men = .79 (n.s.); criteria variable **BDI II** & predicting variable **GEPAQ** (**masculine** pt) b-coefficient women = -1.72 (n.s.) & b-coefficient **men** = **-4.23** (p ≤ .01); criteria variable BDI II & predicting variable GEPAQ (feminin pt) b-coefficient women = .95 (n.s.) & b-coefficient men = .-24 (n.s.)

**Conclusions:**

Gender-related personality traits are associated with the occurrence and severity of ‘non-typical’ & ‘typical’ depressive symptoms. They should be considered in the diagnosis & treatment of depression. Gender differences have also been identified. In men, the expression of masculine personality traits does not lead to an increase in depressive symptoms, especially not in ‘non-typical’ depressive symptoms, as was originally thought. Based on these and other recent findings, the concept of MD can be critically discussed.

**Disclosure of Interest:**

None Declared

